# Updates on Hydrogen Value Chain: A Strategic Roadmap

**DOI:** 10.1002/gch2.202300073

**Published:** 2023-07-08

**Authors:** Julio Garcia‐Navarro, Mark A. Isaacs, Marco Favaro, Dan Ren, Wee‐Jun Ong, Michael Grätzel, Pablo Jiménez‐Calvo

**Affiliations:** ^1^ Stichting New Energy Coalition Nijenborgh 6 Groningen 9747 AG The Netherlands; ^2^ Department of Chemistry University College London 20 Gower Street London WC1H 0AJ UK; ^3^ HarwellXPS Research Complex at Harwell Rutherford Appleton Lab Didcot OX11 0FA UK; ^4^ Institute for Solar Fuels Helmholtz‐Zentrum Berlin für Materialien und Energy GmbH Hahn‐Meitner‐Platz 1 14109 Berlin Germany; ^5^ School of Chemical Engineering and Technology Xi'an Jiaotong University West Xianning Road 28 Xi'an 710049 China; ^6^ School of Energy and Chemical Engineering Xiamen University Malaysia Darul Ehsan Selangor 43900 Malaysia; ^7^ Center of Excellence for Nano Energy & Catalysis Technology (CONNECT) Xiamen University Malaysia Darul Ehsan Selangor 43900 Malaysia; ^8^ State Key Laboratory of Physical Chemistry of Solid Surfaces College of Chemistry and Chemical Engineering Xiamen University Xiamen 361005 China; ^9^ Shenzhen Research Institute of Xiamen University Shenzhen 518057 China; ^10^ Laboratory of Photonics and Interfaces Institute of Chemical Sciences and Engineering École Polytechnique Fédérale de Lausanne (EPFL) Lausanne 1015 Switzerland; ^11^ Department of Colloid Chemistry Max‐Planck‐Institute of Colloids and Interfaces Am Mühlenberg 1 14476 Potsdam Germany; ^12^ Present address: Department of Materials Science WW4‐LKO University of Erlangen‐Nuremberg Martensstraße 7 91058 Erlangen Germany

**Keywords:** consumption, hydrogen, production, storage, transport, value chain

## Abstract

A strategic roadmap for noncarbonized fuels is a global priority, and the reduction of carbon dioxide emissions is a key focus of the Paris Agreement to mitigate the effects of rising temperatures. In this context, hydrogen is a promising noncarbonized fuel, but the pace of its implementation will depend on the engineering advancements made at each step of its value chain. To accelerate its adoption, various applications of hydrogen across industries, transport, power, and building sectors have been identified, where it can be used as a feedstock, fuel, or energy carrier and storage. However, widespread usage of hydrogen will depend on its political, industrial, and social acceptance. It is essential to carefully assess the hydrogen value chain and compare it with existing solar technologies. The major challenge to widespread adoption of hydrogen is its cost as outlined in the roadmap for hydrogen. It needs to be produced at the levelized cost of hydrogen of less than $2 kg^−1^ to be competitive with the established process of steam methane reforming. Therefore, this review provides a comprehensive analysis of each step of the hydrogen value chain, outlining both the current challenges and recent advances.

## Graphical abstract

This review outlines the hydrogen (H_2_) value chain: production, transport, storage, and consumption. Green and white H_2_ holds promise for the future. Repurposing natural gas infrastructure and exploring H_2_ shipping methods pose challenges and opportunities. Salt caverns offer storage potential. H_2_ is crucial in industry, mobility, and the built environment, but faces challenges in social acceptance and transport infrastructure.

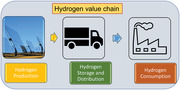

## 1 Introduction

Decarbonization, strive for net zero emissions, and work to reduce our carbon footprint. These crucial actions are indispensable in addressing the climate change challenges. By embracing decarbonization strategies and adopting sustainable practices, we can alleviate the detrimental impacts of greenhouse gas emissions and protect the well‐being of the planet and future generations.^[^ ^1^ ^]^

Paris agreement in 2015 introduced the “national low‐carbon strategy” and has emerged as a vital instrument. This ambitious roadmap not only serves as a guiding light but also forms the bedrock for our collective endeavor to foster a low‐carbon economy. The strategy framework delineates a path that advocates for the adoption of energy‐efficient and the utilization of renewable energy sources.^[^ ^1^, ^2^ ^]^

In this context, hydrogen (H_2_) is expected to play a significant and central role in our future society, particularly within the current efforts to transition to a low‐carbon economy.^[^ ^3^ ^]^ The main reasons why H_2_ is becoming such an important commodity are:

- H_2_ is a clean energy source, producing no greenhouse gas (GHG) emissions when used in fuel cells to produce electricity. This makes it an attractive alternative to fossil fuels.
- H_2_ is a versatile energy carrier that can be used in a variety of applications, from fuel cells for transportation to power/heat/electricity generation and industrial processes, e.g., fertilizers.
- H_2_ can be used as an energy storage medium, allowing excess energy from intermittent renewable sources such as solar and wind to be stored efficiently and used when needed.
- The development of a H_2_ infrastructure which encompasses production, storage, and transportation, would create new jobs and economic opportunities in the energy sector.
- The use of H_2_ in transportation and other applications can support the efforts reducing GHG emissions, thereby mitigating the impact of climate change.

**Figure 1:**
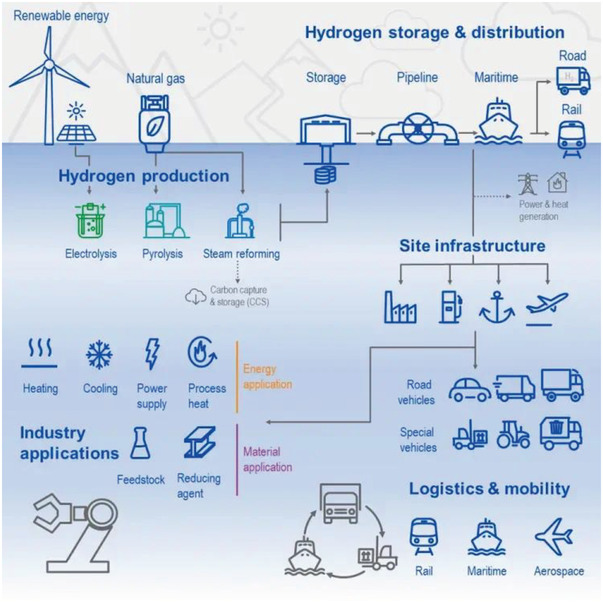
Scheme outlining the hydrogen value chain fields, interconnection, and roles. Reproduced with permission.^[^ ^4^ ^]^ Copyright 2023, TÜV SÜD.

As described previously, the H_2_ value chain consists of three primary areas: production, storage, and distribution (transport) that enable end‐user consumption. **Figure** **1** depicts the layout of the aforementioned fields and their interconnectivity. Yet, each stage faces unique barriers to overcome. Experts from different fields are exploring solutions to provide timely technological transfer options.

The global market demands certain maturity of emerging technologies to deliver them to the end users. Within the hydrogen economy context, the different aspects of the value chain should achieve technological readiness level (TRL) 7 or higher, to make H_2_ and its infrastructures economically (and politically) attractive.

The following sections will showcase each category of the H_2_ value chain, including the main associated challenges.

## 2 Hydrogen Production

Hydrogen production has gained an enormous momentum over the last few years and will certainly play a major role in the energy transition. The reasons behind the growing importance of H_2_ for our modern economy are: 1) decarbonization of energy production, in particular electricity, 2) sustainable alternative to fossil energy carriers (in particular natural gas), and 3) the lack of cost‐effective energy storage of intermittent renewable energy (mostly produced in the form of electricity).

There are three main families of H_2_ production technologies that are categorized by their “carbon‐intensity,” i.e., the amount of CO_2_ and CO_2_‐equivalents emitted during production of H_2_. These are described in the following sub‐sections in descending order of carbon‐intensity.

### 2.1 Grey hydrogen

Grey H_2_ is produced by dehydrogenation of fossil fuels. There are two grey H_2_ production processes:

1. Reforming: H_2_ is produced by reacting a fossil fuel with steam, thereby releasing H_2_ as well as carbon monoxide, a mixture commonly referred to as “syngas”
(1) $$\begin{array}{c} C_{m}H_{n}+mH_{2}O\to m\mathrm{CO}+\left(m+n/2\right)H_{2} \end{array}$$
2. Partial oxidation: in this process, a fossil fuel is combusted with a sub‐stoichiometric amount of oxygen, leading to the production of syngas
(2) $$\begin{array}{c} C_{m}H_{n}+m/2O_{2}\to m\mathrm{CO}+n/2H_{2} \end{array}$$

The stoichiometric factors for reforming and partial oxidation (Equations (1) and (2) are 1 for *m*, and 4 for *n*.

Steam methane reforming (SMR) is the leading process to produce grey H_2_. In 2021, the global H_2_ demand reached a peak of 94 Mt.^[^ ^5^ ^]^ Currently, about 48% of the H_2_ produced worldwide (≈45 Mt in 2021) comes from SMR.^[^ ^6^, ^7^ ^]^ This process consists of dehydrogenating methane (commonly from a natural gas stream) to produce a hydrogen‐rich mixture, and it is the cheapest at the industrial scale. However, its environmental impact is very high: every kg of H_2_ produced in this way generates around 8 kg of CO_2_, indirectly.^[^ ^7^ ^]^ Oil and coal gasification are still heavily used, accounting for the 30% (≈28 Mt) and 18% (≈17 Mt) of the world's H_2_ production in 2021, respectively.^[^ ^7^ ^]^ Typically, grey H_2_ production processes include additional steps (e.g., water–gas shift and acid gas removal) to maximize the H_2_ yield and separate the carbon oxides from the H_2_ stream.

Among the traditional methods of producing grey H_2_ (reforming and partial oxidation), autothermal reforming (ATR) is used in less proportion. ATR consists of the reforming of light alkanes (typically methane) using purified oxygen and carbon dioxide or water steam. Due to the exothermic character of reactions occurring during methane ATR, the overall process has the ability of self‐maintaining the reaction temperature, thereby approaching zero net enthalpy.^[^ ^8^ ^]^

### 2.2 Blue Hydrogen

The production process of blue H_2_ is similar to that of grey H_2_; the main difference is that blue H_2_ includes the carbon capture and either utilization or storage (CCUS). The capture of carbon consists of the separation of CO_2_ from a mixture (e.g., a flue gas from a combustion process or a CO_2_‐rich natural gas stream) via physical or chemical means. The separation of carbon from the fuel is typically done using one of three methods:

- Precombustion—the carbon is separated from the fuel before being combusted, as in analogy to the SMR process;
- Postcombustion—the carbon is separated after the fuel has been used to generate energy; and
- Oxyfuel combustion—this technology is similar to postcombustion, but oxygen is used instead of air to combust the carbon‐rich fuel, leading to a more complete combustion that releases less CO and has an inherently higher efficiency.

Although there are different processes to separate the resulting CO_2_, most used carbon capture processes rely on postcombustion technologies. **Table** **1** showcases how postcombustion carbon capture is performed, as well as the advantages and challenges of each technology.^[^ ^9^ ^]^

**Table 1:** Overview of the CO_2_ separation technologies

CO_2_ separation method	Description	Strengths	Challenges	Ref.
Absorption	CO_2_ is absorbed in a fluid (called absorbant) that selectively binds to the CO_2_. The CO_2_ is then released from the absorbant (regeneration) Typical absorbants: monoethanolamine (MEA)^[^ ^9^ ^]^	Mature technology, has been in use for 100 years; also known as “amine sweetening,” “acid gas removal” or “amine gas treating” (used to remove H_2_S and CO_2_ from natural gas) Good CO_2_ selectivity High absorption rate Easy to retrofit existing plants to include it	Absorbant regeneration is energy‐intensive and costly for the recovery Corrosiveness of absorbent Loss of solvent Involving toxic chemicals (aliphatic amines)	[9, 10, 11, 12]
Adsorption	CO_2_ is adsorbed on the surface of a highly porous solid (called adsorbent) and later released from it (regeneration) Typical adsorbents: activated carbon, zeolites, metal‐organic frameworks, carbon nanotubes	Lower energy consumption to release CO_2_ from adsorbent	Adsorbent regeneration is energy‐intensive Costly adsorbent Low mass production of adsorbent No widespread industrial applications yet (moderate TRL)	[9, 10, 13, 14]
Biological	Use of photosynthetic organisms for CO_2_ capture. Algae have better carbon stabilization than land plants	It preserves healthy lands, water, and air	Costly The amount of land required to scale up this technology might be high	[9, 10, 15, 16]
Other technologies	Membrane separation – uses a membrane that is CO_2_‐permeable Chemical looping – using a chemical (e.g., a metal oxide) to form carbonates, which can later be used to produce methane	Lower energy consumption Easier to adapt than other CO_2_ separation technologies	Scaling‐up might be difficult.	[9, 10, 17, 18]

Carbon storage (or sequestration) is the process where the separated CO_2_ is injected in an underground geological formation. An important characteristic of this process is that CO_2_ is stored as a supercritical fluid (the critical point of CO_2_ is at 31 °C and 73 bar). Therefore, not all geological sites are suitable to store the injected CO_2_; often, depleted oil and gas wells are used (or at least considered), due to their ability to hold pressurized fluids. The main challenge of operating pressurized gas wells is to ensure that the gas will not escape either by permeation to the nearby soil or by displacement by another fluid (such as water). Here are a few important properties that ideal CO_2_ storage reservoirs^[^ ^19^ ^]^ should present:

- Depth—it is recommended that the wells are at least 1 km underground to ensure that CO_2_ stays in the supercritical phase (i.e., to keep the pressure);
- High capillary pressure—to prevent another fluid from entering the well; and
- Low permeability—to prevent CO_2_ from leaving the well.

The main challenge of blue H_2_ (and CCUS) is primarily the social acceptance of this technology. Society views CCUS as “sweeping the problem under the rug:” CCUS may not constitute a long‐term carbon abatement method and it is simply viewed as “greenwashing” done by the oil and gas industry to maintain the current levels of fossil fuel dependency.

### 2.3 Green Hydrogen

**Figure 2:**
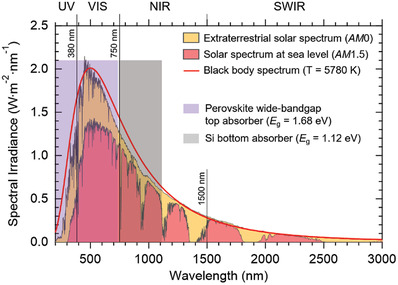
AM0^[^ ^27^ ^]^ and AM1.5^[^ ^28^ ^]^ solar spectra, together with the portion of the spectrum accessible by a double junction tandem perovskite/Si solar cell. In this example, the hybrid organic/inorganic perovskite is the wide‐bandgap top absorber, with a bandgap of 1.68 eV^[^ ^23^ ^]^ (cutoff at ≈740 nm). Single crystalline, n‐type silicon is the narrow‐bandgap bottom absorber, with a bandgap of 1.12 eV at room temperature ^[^ ^23^ ^]^ (cutoff at around 1107 nm). AM: Air Mass; UV: Ultraviolet radiation; VIS: Visible radiation; NIR: Near‐infrared radiation; SWIR: short wavelength infrared radiation.

Green H_2_ is produced by using water and sunlight (or renewable‐driven electricity). There are mainly three types of solar‐driven water electrolysis processes: photocatalytic (PC) water splitting, photoelectrochemical (PEC) water splitting, and photovoltaics (PV) combined with electrolysis.^[^ ^20^, ^21^ ^]^ Among the above three approaches, PV‐driven water splitting already reaches relatively high TRLs (TRL 7), with recently demonstrated solar‐to‐hydrogen (STH) efficiencies up to about 20%.^[^ ^22^ ^]^ Higher efficiencies are foreseen in the near future using tandem solar cells based on improved hybrid organic‐inorganic perovskite light absorbers. Recently, the certified solar to electric power conversion efficiency (PCE) tandems of lead halide perovskite with silicon has reached 32.5%,^[^ ^23^, ^24^ ^]^ a value previously achieved only by expensive III/V semiconductors. **Figure** **2** reports the extraterrestrial/sea level solar spectra (*AM*0 and *AM*1.5), together with the portion of the spectrum accessible by a double junction, tandem perovskite/Si cell (see Figure 2 caption for details). The integration of the *AM*1.5 spectrum up to the near infrared (NIR) cutoff (1107 nm) set by the Si bottom absorber returns an accessible photon flux of about 2.46 × 10^21^ s^−1^ m^−2^. Given an PCE of 30%, the photon flux effectively converted into electricity (≈7.38 × 10^20^ s^−1^ m^−2^) generates a current density of almost 120 A m^−2^, provided by the PV module to the electrolyzer (for this estimation, the PCE was considered equal to 30% for both absorbers in the tandem device). Hence, there is the need to develop electrocatalysts able to provide current densities of about 20 mA cm^−2^ with minimal overvoltage losses, i.e., less than 0.3 V for extended periods of time (months or years), using cheap and earth‐abundant elements.^[^ ^25^, ^26^ ^]^ The water electrolyzer therefore represents the under‐developed component of this approach.^[^ ^4^ ^]^

Catalytic water splitting using photons or electricity occurs in a single step, as Equation (3) shows

(3) $$\begin{array}{c} H_{2}+1/2O_{2}\to H_{2}O \end{array}$$

Water electrolysis processes have been extensively studied and reviewed in recent years, hence a vast amount of literature reviewing the processes already exists.^[^ ^29^, ^30^ ^]^

**Figure 3:**
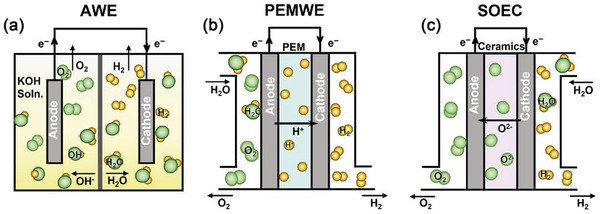
Schemes of a) alkaline water electrolyzer (AWE), b) proton exchange membrane water electrolyzer (PEMWE) and c) solid oxide electrolysis cell (SOEC). Reproduced with permission.^[^ ^95^ ^]^ Copyright 2022, SciOpen.

**Figure 4:**
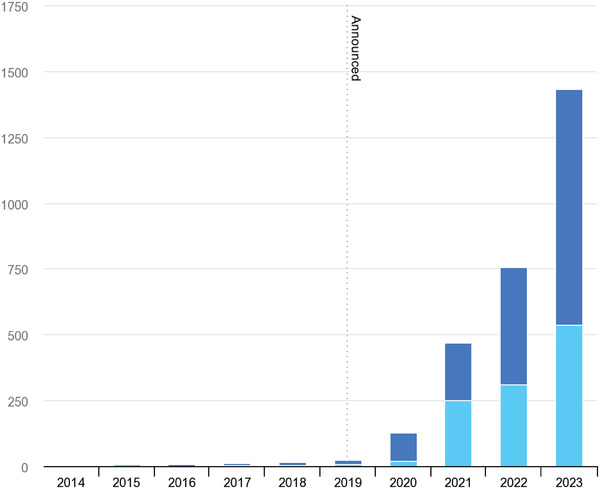
Global electrolysis capacity (MW year^−1^) becoming operational annually, 2014–2023, historical and announced. Dark blue stands for total and light blue stands for largest project. Reproduced with permission.^[^ ^33^ ^]^ Copyright 2022, IEA.

Here we will only highlight the common production methods of green H_2_, that differ in the type of charge carrier that completes the electric circuit of the electrolytic cell (**Figure** **3**):

- Alkaline electrolysis—the charge carrier is ^−^OH. This is the most common process for producing high‐purity H_2_ for uses such as in the food industry (e.g., in the hydrogenation of triglycerides and fatty acids for human consumption) as well as in the current landscape of electrolytic H_2_ production for use as energy carrier.^[^ ^31^ ^]^ Alkaline electrolyzers have been in use since the space age (where their main use was to electrolyze water to produce O_2_ for the astronauts) and they are a well‐developed technology.
- PEM electrolysis—the charge carrier is H^+^. It has been developed in the past few decades using the existing knowledge from PEM fuel cells (as they share the same ion‐conducting polymer membrane). PEM electrolyzers use platinum group metals (PGM) as catalysts. This means that their widespread adoption could burden the supply chain of some components. However, they have an advantage over alkaline electrolyzers in their ability to quickly ramp‐up and ramp‐down to cope with the intermittency of renewable energy systems (RES). Thus, PEM electrolyzers are more suitable for direct connection to renewable electricity than alkaline electrolyzers. This eliminates the need to rely on the existing electricity transport infrastructure.
- Solid oxide electrolysis (SOEC)—the charge carrier is O^2−^. This is a relatively recent development that incorporates progress achieved in the solid oxide fuel cell (SOFC) industry. Whereas both alkaline and PEM electrolyzers operate at near‐ambient conditions, SO electrolyzers operate at temperatures above 700 °C, which implies that they are less suitable for dynamic operation. Furthermore, SO electrolyzers are at an earlier stage of development due in part to a similar status of SOFC technologies. A common challenge for all types of devices described above, is the current lack of large‐scale manufacture of electrolyzers, compared to the current ambitions and targets towards 2030 and beyond. As of 2020, the total worldwide electrolyzer manufacturing capacity was 20 MW/year (**Figure** **4**), mostly due to electrolyzers being hand‐manufactured and tailored to individual customers. According to the European strategy on H_2_ drafted by the European Union (EU) in 2020, the plan is to reach 8.2 GW of electrolyzers by 2030.^[^ ^32^ ^]^ The current disparity between the electrolyzers manufacturing capacity and the ambitious set targets can also be regarded as a potential windfall, given that the European interest in H_2_ could provide for a more certain panorama for private capital to invest in electrolyzer manufacture.

PC and PEC‐based water splitting have lower efficiencies (<1 and 10%, respectively) compared to the PV‐driven process.^[^ ^3^, ^34^ ^]^ However, PC and PEC water splitting are performed using only a catalyst in powder (as suspension or adhere to an electrode), water, and light, thereby making it a revolutionary technology due to its simplicity.^[^ ^35^, ^36^ ^]^ In fact, PC water splitting is considered the “holy grail” reaction in physical chemistry, as it can directly generate H_2_ from water. This is a significant advantage, as it enables the production of a solar fuel without the need for external electricity, resulting in lower operating expenses (OPEX) costs.

**Figure 5:**
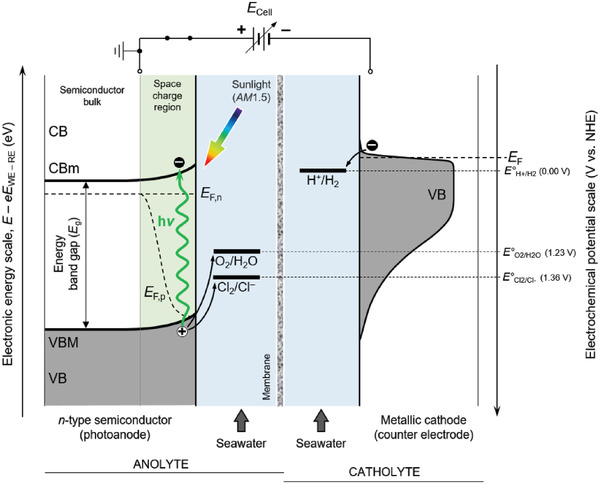
Schematic energy diagram of an H‐type photoelectrochemical cell equipped with a single‐junction semiconducting photoanode absorbing green light (≈495 nm, 2.5 eV) immersed in seawater, under nonequilibrium conditions. Note that the energy axes are not on scale (CB: conduction band; CBm: conduction band minimum; VB: valence band; VBM: valence band maximum; *E*_F_: Fermi level; *E*_F,n_: Quasi Fermi level for electrons; *E*_F,p_: Quasi Fermi level for holes; U: applied potential).

Photocatalysis by definition is a heterogeneous type of catalysis, requiring photons with adequate energy (see **Figure** **5**) to activate the solid semiconductor/catalyst and split water into H_2_ gas.^[^ ^37^, ^38^, ^39^, ^40^ ^]^ Extensive research efforts have been dedicated to the development of binary and multinary metal oxide light absorbers for such applications.^[^ ^41^, ^42^, ^43^, ^44^, ^45^, ^46^, ^47^ ^]^ This class of materials offers a wide range of optical and electronic properties, and generally shows good stability in aqueous environments.^[^ ^48^ ^]^ Combined with a relatively wide bandgap, this makes semiconducting transition metal oxides particularly suited as top absorbers in tandem devices. This is a promising field to explore, since for oxides containing two metal cations about 19 000 different compositions are possible,^[^ ^48^ ^]^ only a small fraction of which has been explored to date. The highest efficiency reported for a bias‐free (unassisted) tandem device with a silicon PV cell providing the lacking photovoltage along with a combination of BiVO_4_ and Fe_2_O_3_ photoanodes achieved *≈*8%. Solar to hydrogen energy conversion efficiency.^[^ ^49^ ^]^

The use of PC and PEC reactions has shown promising results in producing fuels such as H_2_ or alcohols, as well as various chemical products including fine chemicals, pharmaceuticals, and agrochemicals. However, for these processes to become a viable alternative to fossil fuels, they must overcome significant challenges such as maturity, scalability, catalyst efficiency, reactor engineering, and cost.^[^ ^50^ ^]^

PC and PEC have a number of technical challenges to address, such as low photocatalytic activity, large band gap energy, fast recombination of photo‐generated electrons and holes, stability problems, poor light utilization, and cost.^[^ ^51^ ^]^ Overcoming them requires rethinking current tendencies, concepts, and methods to enhance the understanding of the fundamentals of the technology.

Developments at the interface of materials science and engineering of chemical processes are envisaged. On the one hand, steps forward in each low (laboratory reactors), medium (pilot devices), and large (plants) TRLs are mandatory to foster larger H_2_ photoproduction plants.^[^ ^3^, ^52^, ^53^ ^]^ On the other hand, rational design of efficient and stable photoabsorber/catalyst with intriguing aforementioned properties is a high demand task. The importance of this point is to consider all the ecofriendly policies and green chemistry principles to follow decarbonization guidelines. This will not be an easy task, but efforts are being made to satisfy the technological transfer demands with low CO_2_ footprint materials synthesis and processes.

Given the relatively low efficiency of water splitting based on PC and PEC, these approaches should be integrated and adapted to suit specific needs, for instance for the decentralized production of H_2_. In this context, direct seawater splitting is a promising technology,^[^ ^54^, ^55^ ^]^ owing to the abundance and availability of seawater: about 71% of the Earth's surface is water‐covered, with saline water accounting for 96.5% of all the water available on Earth.^[^ ^56^ ^]^ Moreover, 77% of all countries worldwide have direct access to seawater, a renewable resource that is constantly replenished by the natural water cycle. The use of seawater for the decentralized production of H_2_ could eliminate the need to use purified water, which is energy‐intensive to produce. Moreover, in the current scenario of water scarcity worldwide, purified, potable water is a critical commodity: about one‐in‐four people (≈2 billions) do not currently have access to clean water.^[^ ^57^ ^]^ Unfortunately, this situation might worsen in the near future, due to the global warming and related climate change. Direct seawater splitting has the potential to be independent from energy‐intensive centralized desalination facilities, which should be prioritized toward the production of purified water for human consumption and use in agriculture. Therefore, direct seawater splitting could constitute a sustainable and readily available option for the de‐centralized, local generation of green H_2_, while avoiding competing with the production of potable water. Cheap and Earth‐abundant oxide‐based semiconducting light absorbers could be pivotal for this technology: the main advantage of direct PC/PEC seawater splitting is that it does not require external sources of electricity to sustain the electrolysis, as schematically reported in Figure 5 for a single junction device. Instead, the process is powered directly by sunlight, which is free, abundant (1.0 kW m^−2^ at *AM*1.5), and renewable. In addition, due to the high concentration of NaCl in seawater (≈0.5 mol L^−1^),^[^ ^58^ ^]^ this process could provide chlorine gas as a side (anodic) product, a commodity used to disinfect water, for the sanitation of sewage and industrial waste, and for production of derived chemicals and polymers. This side production could add a significant contribution to the value chain of H_2_.

**Figure 6:**
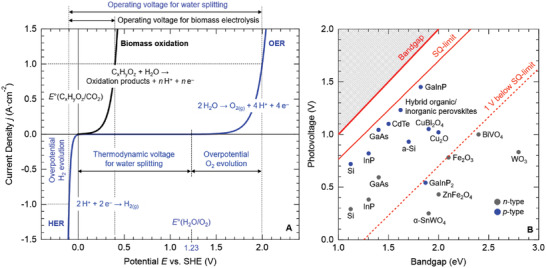
A) Theoretical reaction kinetics controlled by the Butler–Volmer law for water splitting and the electrolysis involving biomass oxidation. For both cases the operating voltages have been determined for a current density of 1 A cm^−2^. Adapted from C. Lamy, C. Coutanceau, S. Baranton, Production of Clean Hydrogen by Electrochemical Reforming of Oxygenated Organic Compounds, Academic Press, Cambridge, Massachusetts 2020.^[62]^ B) Photovoltage as a function of bandgap for selected semiconductors. The Shockley–Queisser (SQ) limit is also indicated. Adapted from Matthew T. Mayer, Photovoltage at semiconductor–electrolyte junctions, Curr. Opin. Electrochem., 2017, 2, 104–110 (themed issue on Solar Cells edited by Michael Grätzel). HER: Hydrogen Evolution Reaction; OER: Oxygen Evolution Reaction.

The aforementioned green H_2_ production methods face the common challenge of high energy costs required to split water. According to thermodynamics, the energy input required for this reaction is equivalent to an applied voltage of 1.23 V. However, in practice, a voltage of more than 1.7 V must be applied to overcome the sluggish kinetics of the oxygen evolution reaction (OER),^[^ ^59^ ^]^ which is the bottleneck of water splitting. Even the most efficient OER electrocatalysts to date show significant overpotentials (up to 0.4 V),^[^ ^59^ ^]^ which leads to a significant amount of energy being spent on producing oxygen, a product with low market value. As a result, the deployment of the green H_2_ production market is hindered: green H_2_ currently accounts for only 4% of the overall production worldwide, equivalent to 3.8 Mt in 2021.^[^ ^6^ ^]^ Interestingly, this is in line with the amount of electricity produced worldwide using solar PV, accounting for 3.6% (179 TWh in 2021).^[^ ^60^ ^]^ Therefore, boosting the large‐scale production of green H_2_ requires the parallel optimization and scale‐up of both solar PV and electrolyzers. Value‐added oxidation reactions are an attractive pathway to further reduce green H_2_ production costs by reducing energy consumption and adding market value.^[^ ^59^ ^]^ In this class of reactions, electrolysis is performed in aqueous solutions containing biomass‐derived waste analogs such as glycerol or 5‐hydroxymethylfurfural (5‐HMF), which are used as efficient electron donors. Biomass electrolysis offers a potential solution to replace the sluggish OER at the anodic side of (photo)electrolyzers with the more favorable organic molecule oxidation, which could lead to a more efficient production process.^[^ ^59^ ^]^ The H_2_ produced in this way is still considered “green” since biomass is recognized as a CO_2_‐neutral, abundant, and renewable substitute for fossil fuels.^[^ ^61^ ^]^ Additionally, the rich proton content in most biomass building blocks makes it an effective H_2_ carrier.^[^ ^59^ ^]^ Compared to water electrolysis, the electrolysis of biomass feedstock to generate H_2_ requires less theoretical electricity consumption, as previously reported^[^ ^62^ ^]^ and as schematized in **Figure** **6**a. Noteworthy, the theoretical operating voltage for a device performing biomass electrolysis could be as low as 0.5 V. From a PC/PEC point of view, this voltage could be achieved with the photovoltage generated by a double‐or even a single junction photoabsorber system, as reported in Figure 6b. This could constitute a net advantage over water electrolysis, since the higher operating voltages required to split water necessitate multiabsorber tandems connected in series to yield additive photovoltages. Furthermore, biomass oxidation offers the opportunity to produce value‐added chemicals, representing a highly efficient power‐to‐X (X = fuel and chemicals) conversion.^[^ ^59^ ^]^ These combined aspects have the potential to produce green H_2_ at relatively low cost, adding further value by the generation of diverse chemical commodities. However, the practical implementation of this promising technology is subject to further investigation and consideration, particularly regarding the true kinetic overpotentials of these reactions and their dependence on reaction conditions, the cost and energy required for the purification of the oxidation products, and the market demand for such commodities.

### 2.4 White Hydrogen

White H_2_, so called natural H_2_, is generated by a natural geochemical process deep inside the Earth's crust. This form of hydrogen production stands as the most cost‐effective method for producing carbon‐neutral H_2_ and is competitive with fossil fuels. In terms of its value chain, the processes involved mirror those employed in natural gas production, encompassing prospecting, site selection, drilling, extraction, and product separation. An important aspect to highlight is the utmost priority placed on environmental protection throughout the implementation of this solution. Notably, the extraction of natural H_2_ circumvents the need for contentious techniques like hydraulic fracturing, ensuring a sustainable and responsible approach.^[^ ^63^, ^64^ ^]^

Among the most impressive advantages of this H_2_ family is that is generated constantly in the Earth's depths and its flux is estimated of 23 million ton of H_2_ per year from all geological sources. Additionally, one kilogram extraction prevents 7.7 kg of CO_2_, therefore the expected price is even cheaper than the steam methane reforming (grey) H_2_, currently in $2 kg^−1^.^[^ ^63^, ^64^ ^]^

## 3 Hydrogen Transport

The existence of a transport and storage infrastructure for H_2_ could kickstart the consumption of H_2_ for all three types of consumers (in particular for mobility and the built environment). Up to this day, the amount of H_2_ consumed outside of industrial facilities is significantly low (mainly used as a reagent in research laboratories, in some captive medium‐duty vehicles (MDV) fleets, and the few available hydrogen refueling stations (HRS) worldwide before 2010). In this context, the development of the (trans)national H_2_ transport networks anywhere has not been felt by stakeholders in the H_2_ sector.

### 3.1 Transport of Hydrogen in the Existing Natural Gas Infrastructure

The most popular method of transporting H_2_ nowadays is via the use of tube trailers, which is merely a trailer that carries a bundle of tubes and supplies gases to a particular consumer. The main disadvantage of transporting H_2_ via tube trailers is the high cost of transported H_2_: a single tube trailer can only carry ≈400 kg of H_2_, and the limitation lies in the weight of the steel tubes used as well as in the maximum allowable storage pressure in tube trailers (250 bar in the U.S.).^[^ ^65^ ^]^

One of the proposals that has recently gained significant attention in the H_2_ world is to repurpose the existing natural gas transmission and distribution infrastructure. Transporting H_2_ in a network that has already been commissioned would bring significant advantages including 1) a short(er) time horizon for the development of a large‐scale H_2_ transport infrastructure, 2) significantly lower capital expenditures (CAPEX) of the transport system for H_2_, and 3) to bring freedom and certainty to stakeholders on both the consumption and production sides of H_2_, so that they can develop projects where they deem it best suited to their interests.

The retrofitting of the existing natural gas infrastructure to accommodate H_2_ comes with challenges of its own. As a first example, there could be competition with natural gas. The International Energy Agency (IEA) predicts that natural gas will continue to play a role in the global energy mix, both in the lead up to 2050 and potentially beyond.^[^ ^66^ ^]^ This implies that H_2_ and natural gas could compete with one another for access to the transport infrastructure; in principle, this could be solved by physically blending H_2_ in the natural gas, albeit blending also comes with its own set of risks (increased flammability of the mixture compared to pure natural gas) and challenges.

Furthermore, compatibility of the natural gas infrastructure with H_2_ can also present a difficult challenge. The development of gas transport infrastructure was historically done in view of two types of gas to deliver: 1) the transport of “town gas,” i.e., gasified coal that consists of a mixture of carbon monoxide and H_2_, in urban settlements, and 2) the natural gas (either low caloric or high caloric) that holds little to no H_2_. As such, it is not expected that H_2_ would be compatible with the existing assets in the natural gas transport infrastructure, in particular looking at the transmission grid (in many countries it consists of large‐diameter pipelines with a nominal operating pressure upwards of 50 bar) and being mindful of pressure‐induced phenomena such as permeation, leaks, or hydrogen‐induced embrittlement.

There are several past and ongoing projects^[^ ^67^, ^68^, ^69^, ^70^, ^71^ ^]^ to prove the extent to which the assets of the natural gas infrastructure can be reused for H_2_ or H_2_ blends and to which extent they would need to be replaced or maintained more often (both of which would result in increased H_2_ transport costs). Research in this topic has converged so far to the knowledge that the existing natural gas infrastructure can carry H_2_ blends up to 20–30% without serious modifications, while blending higher H_2_ percentages as well as carrying 100% H_2_ need more research.

### 3.2 Hydrogen Shipping and Hydrogen Carriers

Hydrogen transportation is a crucial component in establishing a global H_2_ economy. While pipeline transport is practical for short distances, the transportation of H_2_ across oceans is necessary for long distances. The development of H_2_ shipping using various carriers, including compressed gas, liquefied H_2_, and ammonia, presents an opportunity for countries to access clean energy sources that may not be available locally. However, several challenges need to be addressed, such as safety, cost‐effectiveness, and competition with existing supply chains.

This type of transportation is known as H_2_ shipping and can be accomplished using various H_2_ carriers (**Table** **2**).^[^ ^8^ ^]^

**Table 2:** Hydrogen carriers with description, potential windfalls, and challenges

Hydrogen carrier	Description	Potential windfalls	Challenges
Liquid hydrogen	H_2_ is liquefied and transported using tankers to an import terminal. From there, it is transported further inland either as a liquid or in a gaseous state.	The advantage of this approach is that the liquefied natural gas (LNG) trade is already established worldwide. Therefore, by learning from the existing LNG supply chain, the transportation of liquid H_2_ could be further developed.	The primary challenge of liquid H_2_ is boil‐off losses caused by imperfect insulation and quantum effects.
Ammonia	H_2_ is used to synthesize ammonia but, instead of the ammonia becoming the feedstock for a further chemical process (e.g., in the production of fertilizers), ammonia is shipped to a reconversion terminal where H_2_ is extracted from the ammonia and used in gaseous form.	Shipping H_2_ as ammonia can benefit from the established worldwide ammonia trade.	While the Haber‐Bosch process to produce ammonia is well‐established, the reverse reaction to release H_2_ is still in the early stages of development. This suggests that it may take some time to develop this form of shipping. Furthermore, ammonia has been reported as a particularly toxic compound for humans^[^ ^72^ ^]^ Another challenge is the potential competition with the current ammonia and ammonia‐derived supply chains, e.g., fertilizers. This suggests that using hydrogen as an energy carrier could potentially compete with food production.
Liquid organic hydrogen carrier (LOHC)	An unsaturated hydrocarbon (such as methyl cyclohexane, *n*‐ethyl carbazole, and dibenzyl toluene) is hydrogenated to form a saturated hydrocarbon (typically in liquid form), and is later transported to a reconversion terminal where it is dehydrogenated, and the residual liquid (i.e., an unsaturated hydrocarbon) is shipped back to the origin	The use of LOHC can benefit from the existing worldwide trade (in particular of toluene, the dehydrogenated form of methyl cyclohexane) Unlike liquid H_2_, LOHCs can be stored in liquid form at ambient conditions and, unlike ammonia, LOHCs do not pose a particularly high threat to human health	Whereas ammonia and liquid H_2_ are considered “one‐way carriers”, i.e., they get destroyed after extracting the H_2_, LOHCs are meant to be two‐way carriers, meaning that the dehydrogenated liquid will be shipped back to the port of origin for re‐hydrogenation. This means that LOHCs are probably more suitable for short distance transport, e.g., between the mainland and an island, instead of being an optimal candidate for transoceanic trade. Both the de‐ and the hydrogenation of LOHCs processes are not currently done at a large scale in the world and are currently at a developmental stage, meaning that LOHC could come to the market later than the alternative H_2_ carriers

Table 2 is not exhaustive. There are other H_2_ carriers at various development stages (such as methanol, alkali borohydrides, and metal hydrides),^[^ ^73^ ^]^ however, the three H_2_ carriers previously discussed are the most often mentioned in the H_2_ community.

In summary, the benefits of establishing a global H_2_ economy, including reducing greenhouse gas emissions, enhancing energy security, and fostering international collaboration, outweigh the challenges and make the development of H_2_ transportation a priority for a sustainable future.

## 4 Hydrogen Storage

An important component of the supply chains of fossil fuels is the storage facilities. This is particularly relevant for gaseous fuels such as natural gas, where large‐scale storage requires large volumes that can only be found underground. Storage of energy carriers is vital to the social acceptance, immediate implementation, and economy inclusion. It is also the primary method of coping with the fluctuations in supply and demand. One of the causes of the spike in the natural gas price at the end of 2021 was indeed the depletion of the European gas reservoirs that were put under stress by the decreased natural gas supply from abroad, namely Russia.

Underground storage of H_2_ can be done in a similar fashion as the underground storage of natural gas, i.e., in salt caverns. Salt caverns are synthetic subsurface structures that are developed by “washing” away salt from an underground salt deposit until a hollow structure is formed. A salt cavern has a maximum depth of 2000 m, a volume of 1 000 000 m^3^, a height ranging from 300 to 500 m, and a diameter between 50 and 100 m. These dimensions enable substantial storage capacity within a pressure range of 30 to 80% of the lithostatic pressure. Nevertheless, when a well‐designed cavern is constructed, salt deformation can still arise at depths exceeding 2000 m as a result of elevated pressure and temperature.^[^ ^74^ ^]^ Salt caverns offer the most promising underground storage option due to the large sealing capacity of rock salt, its inert nature, as well as their flexibility toward injection/withdrawal cycles.^[^ ^75^, ^76^ ^]^ For further details on operational storage conditions, which depends on the cavern dimensions, refer to the review of Muhammed et al.^[^ ^74^ ^]^

**Figure 7:**
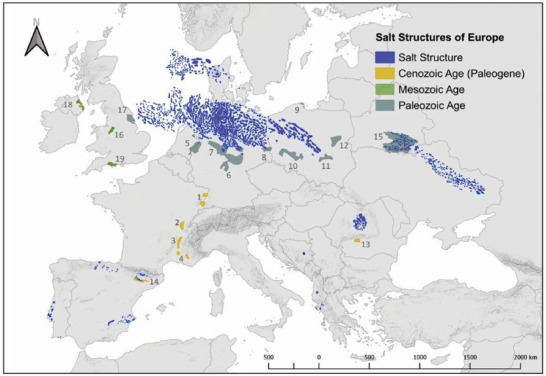
Salt structures localized along Europe. Reproduced with permission.^[^ ^75^ ^]^ Copyright 2020, Elsevier.

Although salt deposits are not commonplaces in the world, they are not particularly rare, either: there is a significant amount of salt deposits spread throughout Europe (**Figure** **7**). It is estimated that the total on‐ and off‐shore European H_2_ storage potential is 84.8 PWh_H2_,^[^ ^75^ ^]^ which represents more than 8 times of the total final energy consumption in Europe in 2020.^[^ ^77^ ^]^ This reinforces the potential to store significant amounts of H_2_ in Europe, enough to cover a potential surge in demand in the coming decades.

The investigation of underground H_2_ storage is currently underway in various regions of the world. However, this research is at an earlier stage compared to other aspects of the H_2_ value chain. Consequently, the deployment of large‐scale H_2_ storage may take more time. Nevertheless, an appealing underground H_2_ storage is the H_2_ project at Teesside, UK.^[^ ^78^ ^]^ British Petroleum and stakeholders aimed at creating the United Kingdom's first fully integrated H_2_ production and CCUS hub. The Teesside project aims to lower the industrial cluster's carbon footprint by generating blue H_2_ from natural gas, capturing the CO_2_, and storing it offshore. The initiative also has economic benefits and supports the UK's net‐zero emissions target.

Another clear example for the same effort is the HyStock project at Zuidwending, the Netherlands.^[^ ^79^ ^]^ This project particularly will investigate the effect of salt cavern storage on the purity of H_2_ and the technical feasibility (especially in safety aspects) of salt cavern storage for H_2_.

Meanwhile, the storage of H_2_ in salt caverns is already implemented on a large industrial scale, its applicability is limited in certain regions due to differing geological conditions. Thus, another storage technologies are necessary for worldwide implementation. Among the developed technologies, H_2_ storage can be classified into two groups based on its physical state: gaseous or liquid. Additionally, H_2_ storage methods can be further categorized as either adsorption, which occurs on the surfaces of solids, or absorption, which takes place within solids. The common physical storage methods are compressed gas, cold/cryo compressed gas, and liquid H_2_. Whilst the materials‐based methods are adsorbent, liquid organic, hydride (interstitial or in complex), and chemical H_2_.^[^ ^80^, ^81^ ^]^ One emerging H_2_ storage form is applying high pressure and preserving the room temperature, several studies have been reported.^[^ ^82^ ^]^

## 5 Hydrogen Consumption

The consumption of H_2_ to produce either (mechanical) work or heat is an important step of the value chain. Although the consumption of H_2_ has been generally associated with the operation of fuel cells, H_2_ can also be burned as any other gaseous fuel to produce heat in a boiler or stovetop as well as combustion engines for cars. H_2_ consumption is generally divided into the types of end‐users:

1. Industry
2. Mobility
3. Built environment

### 5.1 Hydrogen in Industry

Interestingly, according to IEA, industry is the second‐largest global source of CO_2_ emissions (related to the energy sector, after the combustion of fossil fuels), exceeding 8.4 Gt in 2020.^[^ ^83^ ^]^ In particular, three heavy industries, i.e., chemicals (production of primary chemicals, i.e., ethylene, propylene, benzene, toluene, ammonia, and methanol), steel, and cement, account for nearly 60% of all energy consumption and about 72% of all the industrial CO_2_ emissions.^[^ ^66^ ^]^

Electrifying industry has been a viable option for years, but it has two challenges. First, the cost of electricity per unit energy (MWh) is higher than fossil fuels, making it less appealing for businesses. Second, industrialized countries such as Germany, Poland, the US, and the Netherlands produce electricity with high carbon intensity (>300 g CO_2_ kWh^−1^),^[^ ^84^ ^]^ making electrification ineffective in reducing industry's carbon footprint.

In densely populated countries like the Netherlands, the electricity grid is near its maximum transport capacity, causing congestion problems. Large off‐takers and electricity producers joining the network without a planned expansion worsen the situation. Hence, H_2_ is seen as a potential substitute for electrification. In industry, there are two primary methods for utilizing H_2_. The first is to generate heat, primarily high‐temperature heat. The second is to use it as a chemical reagent in processes such as the Haber–Bosch process for obtaining ammonia.

If heat is the main input needed by a particular industry, then in principle H_2_ can directly substitute fossil fuels such as natural gas and coal. However, current developments have pointed to potential challenges about the combustion of H_2_:

- Handling H_2_ safely requires additional attention compared to gaseous fossil fuels such as natural gas. It is necessary to ensure that the safety standards for handling H_2_ are at least as high as those for natural gas.
- Since the flame temperature of H_2_ (2180–2210 °C) is higher than that of natural gas (1930–1960 °C),^[^ ^85^ ^]^ there is a growing concern that burning H_2_ can lead to an increase in (mainly thermal) NOx emissions as a result of the increased flame temperature. In this case, current developments in combustion technology and burner design are underway to solve this problem.

As mentioned before, H_2_ can also be used as feedstock, i.e., as a chemical reagent to take part in the Haber–Bosch process to produce ammonia

(4) $$\begin{array}{c} 3H_{2}+N_{2}\to 2NH_{3} \end{array}$$

Using H_2_ to produce ammonia is a well‐established process. However, it has a disadvantage—H_2_ can also be obtained from natural gas through SMR.^[^ ^86^ ^]^ As a result, substituting H_2_ in ammonia plants is primarily a cost optimization challenge.

H_2_ can be feedstock within the production of steel,^[^ ^87^, ^88^, ^89^ ^]^ namely the direct reduction of iron

(5) $$\begin{array}{c} 3Fe_{2}O_{3}+9H_{2}\to 6\mathrm{Fe}+9H_{2}O \end{array}$$

In a steel production,^[^ ^87^, ^88^, ^89^ ^]^ the challenge is exacerbated by the existing flexibility in the current steel manufacturing processes: iron can be reduced not only with H_2_ but also with CO, involving other types of possibly more polluting fuels such as coke‐oven gas, refinery bottoms, coal, and pet coke, which can be used in the process. Eventually, the introduction of H_2_ to the industry will largely depend on its price, which will be influenced by many parameters, among them the costs of its transportation and production.

### 5.2 Hydrogen in Mobility

Mobility has been the most discussed application of H_2_ in the past decades. Since the surge in interest by US‐based car manufacturers to develop H_2_ cars in the 1990s, H_2_ has ever since been promoted as a substitute for gasoline and diesel (and to a lesser extent, for liquefied petroleum gas, liquefied petroleum gas (LPG), and liquefied natural gas (LNG). As is the case for industrial end‐users looking for a substitute to fossil‐fuel combustion, H_2_ could be used in an internal combustion engine (ICE), although the vehicle would probably need to be adjusted to the H_2_ consumption. The other main applications for extracting energy from H_2_ are PEM fuel cells, considered mainly due to their higher efficiency (≈50%) with respect to internal combustions (ICEs) (≈30%); the operation of PEM fuel cells has already been extensively covered in the literature.^[^ ^90^ ^]^ There are several kinds of mobility applications including light‐duty vehicles (LDVs) (light‐duty vehicles, i.e., passenger cars), medium‐duty vehicles (MDVs) (e.g., vans, public transport buses), and heavy‐duty vehicles (HDVs) like long‐haul trucks and coaches.

Regarding LDVs, fuel cell vehicles have existed in the market for longer than a decade, with Japanese and Korean car manufacturers being the most prominent in the market. The main challenge with respect to LDVs is the availability of HRSs, although the current support for hydrogen‐based mobility has accelerated the HRS market, from 330 stations available in 2017 to 540 in 2020.^[^ ^91^ ^]^ Besides the infrastructure availability, the cost of the LDVs fuel cell and H_2_ as fuel are considered a challenge to be overcome by the development of both suitable transport and storage infrastructure. Undeniably, the governmental policies aimed at abating carbon emissions in mobility are of great importance, too.

The requirements for introducing H_2_ for powering MDVs and HDVs are similar to LDVs, although these vehicles present challenges (in particular for HDVs) due to the more stringent operational requirements with respect to LDVs. For example, while an average LDV might drive ≈20.000 km per year, a long‐haul truck drives at least three times more (≈60.000 km year^−1^) and the overall number of kilometers driven with an HDV before decommissioning is significantly higher than for an LDV. The increased lifetime and operating hours of an HDV mean that the current generation of PEM fuel cells (that were perfected for LDVs as it was the primary driver for their development) cannot follow the more stringent requirements for HDVs (and some MDVs as well). In this context, there are current efforts by companies such as Hyundai, Toyota, and Nikola that seem to bridge these constraints by developing next‐generation PEM fuel cells for heavy duty mobility.

### 5.3 Hydrogen in the Built Environment

The built environment, which comprises households and commercial buildings, is one of the worldwide largest carbon emitters. The Netherlands Environmental Assessment Agency reported in 2019 that the building sector emitted 12% (23.3 MtCO_2_ year^−1^) of the total CO_2_ emissions at the national level.^[^ ^92^ ^]^ Worldwide, almost 24% of the GHG emission is due to the energy production and consumption for the built environment.^[^ ^93^ ^]^

Fossil fuels consumption within the built environment is primarily from cooking, i.e., in stovetops and ovens, or in boiler/heat exchangers to produce space heating and warm water. The challenges for introducing H_2_ into the built environment can be divided in two main categories:

- The social acceptance, where the discussion of safety is centerpiece.
- A robust transport infrastructure to supply households and commercial buildings.

The consumption of H_2_ in the built environment could be technologically feasible in time to come. Boilers and burners could be directly switched over from natural gas to H_2_ with only the addition of a safety device, meant to control the H_2_ flow to the end‐user in case of failure or leak.^[^ ^94^ ^]^

## 6 A Strategic Roadmap on Hydrogen Value Chain

This review provides a comprehensive overview of the key steps and components involved in the value chain of H_2_ production, transportation, and storage, which are essential for the transition to a sustainable energy future. This section aims to offer a concise summary of the main elements within each step, highlighting the significance of H_2_ as a promising noncarbonized fuel.

H_2_ production is gaining momentum due to its role in decarbonizing energy production, providing a sustainable alternative to fossil fuels, and addressing the need for cost‐effective energy storage. There are three leading types of H_2_ production: grey H_2_, which is produced from fossil fuels and has a high carbon intensity; blue H_2_, which includes carbon capture and storage/utilization; and green H_2_, which is produced using water and renewable energy sources. While grey H_2_ is currently the most widely used method, green H_2_ holds promise for the future, but large‐scale electrolyzer manufacturing capacity needs to be developed. One can comment on white H_2_ as perhaps as appealing than green, since its naturally available and the extraction steps are available due to other existent gases infrastructure.

Establishing a transport and storage infrastructure for H_2_ is crucial to promote its consumption, especially in sectors like mobility and the built environment. Repurposing existing natural gas infrastructure can provide advantages such as shorter development time, lower CAPEX, and freedom for stakeholders. However, challenges include competition with natural gas, compatibility issues, and the need for further research on blending H_2_ in the natural gas network. For long‐distance transportation, H_2_ shipping using carriers like liquid H_2_, ammonia, and LOHCs presents opportunities but requires addressing safety, cost‐effectiveness, and competition with existing supply chains. Despite challenges, developing H_2_ transportation is a priority for a sustainable future due to its potential benefits.

Storage facilities are essential for the supply chains of fossil fuels, particularly for gaseous fuels like natural gas, which require large volumes that can only be stored underground. Salt caverns offer a promising option for underground storage of H_2_, as they provide large sealing capacity and flexibility for injection and withdrawal cycles. Europe has significant salt deposits, offering the potential to store substantial amounts of H_2_ to meet future demand. Research on underground H_2_ storage is ongoing, with projects like the Teesside hub in the UK and the HyStock project in the Netherlands exploring the feasibility and benefits of salt cavern storage. One cannot exclude the storage methods at room temperature and high pressures.

H_2_ consumption plays a crucial role in various sectors, including industry, mobility, and the built environment. In the industrial sector, H_2_ can be used as a substitute for fossil fuels in processes requiring heat or as a chemical reagent in reactions such as the production of ammonia and steel. In the mobility sector, H_2_ has been explored as an alternative to gasoline and diesel, either through adjustments to internal combustion engines or by using PEM fuel cells. In the built environment, H_2_ has the potential to replace fossil fuels for cooking and heating purposes, but challenges remain in terms of social acceptance and establishing a reliable transport infrastructure for H_2_ supply.

## 7 Conclusions

In order to attain the energy transition, value chain, technoeconomic studies, and life cycle assessment are key tools to develop and implement synergistically evolving technologies, such as green and white H_2_. Such technologies must be developed with productivity and stability as indicators. At the same time, development of H_2_ technologies should surpass their technical challenges to be able to provide reliable and competent prototype, ready and safe for the end‐user. One of the major challenges is to lower the levelized cost of hydrogen (LCOH) to $2 kg^−1^ to become competitive with fossil sources. Moreover, the performance of the present state‐of‐the‐art green H_2_ production is below par behind the 10% efficiency requirements set by industry. Therefore, extensive works are urgently required to move forward the advancement of renewable energy technologies, specifically the development of large‐scale electrolyzer manufacturing capacity.

In storage options regard, such as salt caverns and physical or materials‐based methods, are being explored to meet future H_2_ demand. The review acknowledges the importance of establishing a transport infrastructure for H_2_ consumption in sectors like mobility and the built environment, addressing challenges related to competition with natural gas and compatibility. It also highlights opportunities and considerations for long‐distance H_2_ transportation using liquid H_2_, ammonia, and LOHCs as carriers. Furthermore, the review emphasizes H_2_’s crucial role in industry, mobility, and the built environment, potentially replacing fossil fuels for heating and cooking. However, challenges remain in terms of social acceptance and establishing a reliable H_2_ transport infrastructure.

## Conflict of Interest

The authors declare no conflict of interest.

## References

[1] I. F. Teixeira , P. Jiménez‐Calvo , Renewable Energy Production and Distribution, Vol. 2, Academic Press, Cambridge, MA 2023, pp. 145–180.

[2] T. M. L. Wigley , Clim. Change 2018, 147, 31.

[3] M. Isaacs , J. Garcia‐Navarro , W.‐J. Ong , P. Jiménez‐Calvo , Glob. Challenges 2022, 2200165. 10.1002/gch2.202200165 PMC10000254 36910466

[4] T. Ü. V. SÜD , Safety and efficiency along the complete hydrogen value chain, https://www.tuvsud.com/en/themes/hydrogen/explore‐the‐hydrogen‐value‐chain, (accessed: June 2022).

[5] International Energy Agency (IEA), Hydrogen – Analysis – IEA, https://www.iea.org/reports/hydrogen, (accessed: March 2023).

[6] E. Taibi , R. Miranda , W. Vanhoudt , T. Winkel , J.‐C. Lanoix , F. Barth , 2018. https://www.irena.org/-/media/files/irena/agency/publication/2018/sep/irena_hydrogen_from_renewable_power_2018.pdf

[7] G. Franchi , M. Capocelli , M. De Falco , V. Piemonte , D. Barba , Membrane 2020, 10, 10. 10.3390/membranes10010010 PMC7022555 31947783

[8] J. J. Lamb , M. Hillestad , E. Rytter , R. Bock , A. S. R. Nordgård , K. M. Lien , O. S. Burheim , B. G. Pollet , In Hydrogen and Fuel Cells Primers, Hydrogen, Biomass and Bioenergy (EDs: J. J. Lamb , B. G. Pollet ), Academic Press, UK, 2020.

[9] A. I. Osman , M. Hefny , M. I. A. Abdel Maksoud , A. M. Elgarahy , D. W. Rooney , Environ. Chem. Lett. 2020, 19, 797.

[10] P. Madejski , K. Chmiel , N. Subramanian , T. Kuś , Energies 2022, 15, 887.

[11] R. Long‐Innes , H. Struchtrup , Cell Rep. 2022, 3, 100791.

[12] K. Han , C. K. Ahn , M. S. Lee , C. H. Rhee , J. Y. Kim , H. D. Chun , Int. J. Greenhouse Gas Control 2013, 14, 270.

[13] F. Raganati , F. Miccio , P. Ammendola , Energy Fuels 2021, 35, 12845.

[14] R. Maniarasu , S. K. Rathore , S. Murugan , 2022, 10.1177/0958305x221093465.

[15] N. Cihan , G. Bharath , A. K. Nadda , O. YukselOrhan , Int. J. Greenhouse Gas Control 2021, 111, 103465.

[16] J. Love , Biochem. Soc. Trans. 2022, 50, 987. 35411379 10.1042/BST20210764 PMC9162456

[17] W. Liu , Y. Cai , M. Luo , Y. Yang , P. Li , Ind. Eng. Chem. Res. 2022, 61, 2882.

[18] A. Rybak , A. Rybak , S. Boncel , A. Kolanowska , W. Kaszuwara , S. D. Kolev , RSC Adv. 2022, 12, 13367. 35520128 10.1039/d2ra01585d PMC9066557

[19] J. Garcia‐Navarro , What is CCUS? – How Carbon Capture plays a role in the hydrogen economy – Hydrogen Central, https://hydrogen‐central.com/what‐is‐ccus‐carbon‐capture‐hydrogen‐economy/, 2021.

[20] X. Chen , C. Li , M. Grätzel , R. Kostecki , S. S. Mao , Chem. Soc. Rev. 2012, 41, 7909. 22990530 10.1039/c2cs35230c

[21] M. Grätzel , Nature 2001, 414, 338. 11713540 10.1038/35104607

[22] J. Gao , F. Sahli , C. Liu , D. Ren , X. Guo , J. Werner , Q. Jeangros , S. M. Zakeeruddin , C. Ballif , M. Grätzel , J. Luo , Joule 2019, 3, 2930.

[23] A. Al‐Ashouri , E. Köhnen , B. Li , A. Magomedov , H. Hempel , P. Caprioglio , J. A. Márquez , A. B. M. Vilches , E. Kasparavicius , J. A. Smith , N. Phung , D. Menzel , M. Grischek , L. Kegelmann , D. Skroblin , C. Gollwitzer , T. Malinauskas , M. Jošt , G. Matič , B. Rech , R. Schlatmann , M. Topič , L. Korte , A. Abate , B. Stannowski , D. Neher , M. Stolterfoht , T. Unold , V. Getautis , S. Albrecht , Science 2020, 370, 1300. 33303611 10.1126/science.abd4016

[24] National Renewable Energy Laboratory (NREL), Best Research‐Cell Efficiency Chart, https://www.nrel.gov/pv/cell‐efficiency.html, (accessed: April 2023).

[25] M. Favaro , W. S. Drisdell , M. A. Marcus , J. M. Gregoire , E. J. Crumlin , J. A. Haber , J. Yano , ACS Catal. 2017, 7, 1248.

[26] J. Yang , J. K. Cooper , F. M. Toma , K. A. Walczak , M. Favaro , J. W. Beeman , L. H. Hess , C. Wang , C. Zhu , S. Gul , J. Yano , C. Kisielowski , A. Schwartzberg , I. D. Sharp , Nat. Mater. 2016, 16, 335. 27820814 10.1038/nmat4794

[27] 2000 ASTM Standard Extraterrestrial Spectrum Reference E‐490‐00 | Grid Modernization | NREL, https://www.nrel.gov/grid/solar‐resource/spectra‐astm‐e490.html, n.d.

[28] National Renewable Energy Laboratory (NREL), Reference Air Mass 1.5 Spectra | Grid Modernization | NREL, https://www.nrel.gov/grid/solar‐resource/spectra‐am1.5.html, n.d.

[29] J. W. Ager , M. R. Shaner , K. A. Walczak , I. D. Sharp , S. Ardo , Energy Environ. Sci. 2015, 8, 2811.

[30] C. C. L. McCrory , S. Jung , I. M. Ferrer , S. M. Chatman , J. C. Peters , T. F. Jaramillo , J. Am. Chem. Soc. 2015, 137, 4347. 25668483 10.1021/ja510442p

[31] M. Bodner , A. Hofer , V. Hacker , Wiley Interdiscip. Rev. Energy Environ. 2015, 4, 365.

[32] E. Commission , Communication from the Commission to the European Parliament, the Council, the European Economic and Social Committee and the Committee of the Regions: EU Biodiversity Strategy for 2030 – Bringing Nature Back into Our Lives, Brussels, 2020.

[33] International Energy Agency, Global electrolysis capacity becoming operational annually, 2014–2023, historical and announced, https://www.iea.org/data‐and‐statistics/charts/global‐electrolysis‐capacity‐becoming‐operational‐annually‐2014‐2023‐historical‐and‐announced, 2022.

[34] P. I. Jiménez‐Calvo , Université de Strasbourg, 2019.

[35] K. Masao , I. Okura , Photocatalysis: Science and Technology, Springer, Berlin/Heidelberg, 2002.

[36] A. Fujishima , K. Honda , Nature 1972, 238, 37. 12635268 10.1038/238037a0

[37] P. Jiménez‐Calvo , V. Caps , V. Keller , Renewable Sustainable Energy Rev. 2021, 149, 111095.

[38] J. Li , P. Jiménez‐Calvo , E. Paineau , M. N. Ghazzal , Catalysts 2020, 10, 89.

[39] R. Marschall , Adv. Funct. Mater. 2014, 24, 2421.

[40] L. Schumacher , R. Marschall , Top Curr. Chem. 2022, 380, 53. 10.1007/s41061-022-00406-5 PMC9587104 36269440

[41] P. Jiménez‐Calvo , C. Marchal , T. Cottineau , V. Caps , V. Keller , J. Mater. Chem. A 2019, 7, 14849.

[42] P. Jiménez‐Calvo , V. Caps , M. N. Ghazzal , C. Colbeau‐Justin , V. Keller , Nano Energy 2020, 75, 104888.

[43] H. El Marouazi , P. Jiménez‐Calvo , E. Breniaux , C. Colbeau‐Justin , I. Janowska , V. Keller , ACS Sustainable Chem. Eng. 2021, 9, 3633.

[44] X. Wang , K. Maeda , A. Thomas , K. Takanabe , G. Xin , J. M. Carlsson , K. Domen , M. Antonietti , Nat. Mater. 2009, 8, 76. 18997776 10.1038/nmat2317

[45] I. F. Teixeira , N. V. Tarakina , I. F. Silva , N. López‐Salas , A. Savateev , M. Antonietti , Adv. Sustainable Syst. 2022, 2100429.

[46] P. Jimenéz‐Calvo , A. Sobolewska , M. Isaacs , Y. Zhang , A. Leforestier , J. Degrouard , S. Rouzière , C. Goldmann , D. Vantelon , P. Launois , M. N. Ghazzal , E. Paineau , ChemRXiv 2023, 10.26434/CHEMRXIV-2023-QCXSH. 38085685

[47] P. Jiménez‐Calvo , L. Michel , V. Keller , V. Caps , ACS Appl. Mater. Interfaces 2021, 13, 61015. 34918899 10.1021/acsami.1c16159

[48] K. Sivula , R. van de Krol , Nat. Rev. Mater. 2016, 1, 15010.

[49] J. H. Kim , J. W. Jang , Y. H. Jo , F. F. Abdi , Y. H. Lee , R. van de Krol , J. S. Lee , Nat. Commun. 2016, 7, 13380. 27966548 10.1038/ncomms13380 PMC5477502

[50] M. Jerigova , Y. Markushyna , I. F. Teixeira , B. Badamdorj , M. Isaacs , D. Cruz , I. Lauermann , M. Á. Muñoz‐Márquez , N. V. Tarakina , N. López‐Salas , O. Savateev , P. Jimenéz‐Calvo , Adv. Sci. 2023, 10, 2300099. 10.1002/advs.202300099 PMC10161101 36815368

[51] W.‐J. Ong , L.‐L. Tan , Y. H. Ng , S.‐T. Yong , S.‐P. Chai , Chem. Rev. 2016, 116, 7159. 27199146 10.1021/acs.chemrev.6b00075

[52] P. Jimenéz‐Calvo , M. J. Muñoz‐Batista , M. Isaacs , V. Ramnarain , D. Ihiawakrim , X. Li , M. Ángel Muñoz‐Márquez , G. Teobaldi , M. Kociak , E. Paineau , Chem. Eng. J. 2023, 459, 141514.

[53] A. Galushchinskiy , R. González‐Gómez , K. McCarthy , P. Farràs , A. Savateev , Energy Fuels 2022, 36, 4625. 35558990 10.1021/acs.energyfuels.2c00178 PMC9082502

[54] D. H. Marin , J. T. Perryman , M. A. Hubert , G. A. Lindquist , L. Chen , A. M. Aleman , G. A. Kamat , V. A. Niemann , M. B. Stevens , Y. N. Regmi , S. W. Boettcher , A. C. Nielander , T. F. Jaramillo , Joule 2023, 7, 765.

[55] P. Farràs , P. Strasser , A. J. Cowan , Joule 2021, 5, 1921.

[56] Water Science School | U.S. Geological Survey, https://www.usgs.gov/special‐topics/water‐science‐school, (accessed: March 2023).

[57] H. Ritchie , M. Roser , Our World Data 2021.

[58] S. Dresp , F. Dionigi , M. Klingenhof , P. Strasser , ACS Energy Lett. 2019, 4, 933.

[59] H. Luo , J. Barrio , N. Sunny , A. Li , L. Steier , N. Shah , I. E. L. Stephens , M. M. Titirici , Adv. Energy Mater. 2021, 11, 2101180.

[60] International Energy Agency (IEA), P. Bojek, Solar PV – Analysis, https://www.iea.org/reports/solar‐pv, (accessed: March 2023).

[61] Y. Li , W. Liu , Z. Zhang , X. Du , L. Yu , Y. Deng , Commun Chem 2019, 2, 67.

[62] C. Lamy , C. Coutanceau , S. Baranton , Production of Clean Hydrogen by Electrochemical Reforming of Oxygenated Organic Compounds, Academic Press, Cambridge, MA 2019, p. 1.

[63] A. Prinzhofer , I. Moretti , J. Françolin , C. Pacheco , A. D'Agostino , J. Werly , F. Rupin , Int. J. Hydrogen Energy 2019, 44, 5676.

[64] V. Zgonnik , Earth‐Sci. Rev. 2020, 203, 103140.

[65] U.S. Department of Energy, “Hydrogen Tube Trailers”, https://www.energy.gov/eere/fuelcells/hydrogen‐tube‐trailers, (accessed: June 2020).

[66] International Energy Agency, Net Zero by 2050 A Roadmap for the Global Energy Sector, 2021.

[67] National Gas, FutureGrid, https://www.nationalgas.com/insight‐and‐innovation/transmission‐innovation/futuregrid, (accessed: March 2023).

[68] U.S. Department of Energy, HyBlend: Opportunities for Hydrogen Blending in Natural Gas Pipelines, https://www.energy.gov/eere/fuelcells/hyblend‐opportunities‐hydrogen‐blending‐natural‐gas‐pipelines, (accessed: March 2023).

[69] Hy4Heat, Hy4Heat: A programme of work to investigate the practicalities of using hydrogen for heat in UK homes and businesses, https://www.hy4heat.info/, (accessed: March 2023).

[70] H21 Programme , H21 North of England: A Gas Network for the 21st Century, https://h21.green/, (accessed: March 2023).

[71] The HIGGS project, Higgs: Hydrogen Implementation Guidelines for Gas Systems, https://higgsproject.eu/, (accessed: March 2023).

[72] Ammonia Poisoning | Environmental Pollution Centers, https://www.environmentalpollutioncenters.org/ammonia/, (accessed: March 2023).

[73] A. Züttel , A. Remhof , A. Borgschulte , O. Friedrichs , Philos. Trans. A: Math. Phys. Eng. Sci. 2010, 368, 3329. 20566514 10.1098/rsta.2010.0113

[74] N. S. Muhammed , B. Haq , D. Al Shehri , A. Al‐Ahmed , M. M. Rahman , E. Zaman , Energy Rep. 2022, 8, 461.

[75] D. G. Caglayan , N. Weber , H. U. Heinrichs , J. Linßen , M. Robinius , P. A. Kukla , D. Stolten , Int. J. Hydrogen Energy 2020, 45, 6793.

[76] D. Stolten , B. Emonts , Hydrogen Science and Engineering: Materials, Processes, Systems and Technology, Wiley‐VCH Verlag, Weinheim, Germany 2016.

[77] Eurostat, Energy statistics – an overview – Statistics Explained, https://ec.europa.eu/eurostat/statistics‐explained/index.php?title=Energy_statistics_‐_an_overview#Final_energy_consumption, (accessed: October 2022).

[78] British Petroleum, H2Teesside UK project, https://www.bp.com/en_gb/united‐kingdom/home/where‐we‐operate/reimagining‐teesside.html, (accessed: October 2022).

[79] G. Company , HyStock – power to hydrogen, https://www.hystock.nl/, (accessed: October 2022).

[80] J. Andersson , S. Grönkvist , Int. J. Hydrogen Energy 2019, 44, 11901.

[81] Hydrogen Storage | Department of Energy, https://www.energy.gov/eere/fuelcells/hydrogen‐storage, n.d.

[82] M. Felderhoff , C. Weidenthaler , R. Von Helmolt , U. Eberle , Phys. Chem. Chem. Phys. 2007, 9, 2643. 17627309 10.1039/b701563c

[83] (IEA) International Energy Agency, Global Hydrogen Review, https://www.iea.org/reports/hydrogen, (accessed: July 2022).

[84] Electricity Maps | Live 24/7 CO₂ emissions of electricity consumption, https://app.electricitymaps.com/map, (accessed: July 2022).

[85] I. Glassman , Combustion, Harcourt Brace Jovanovich, 1987.

[86] M. Steinberg , H. C. Cheng , Int. J. Hydrogen Energy 1989, 14, 797.

[87] I. R. Souza Filho , H. Springer , Y. Ma , A. Mahajan , C. C. da Silva , M. Kulse , D. Raabe , J. Clean Prod. 2022, 340, 130805.

[88] I. R. Souza Filho , Y. Ma , M. Kulse , D. Ponge , B. Gault , H. Springer , D. Raabe , Acta Mater. 2021, 213, 116971.

[89] S. H. Kim , X. Zhang , Y. Ma , I. R. Souza Filho , K. Schweinar , K. Angenendt , D. Vogel , L. T. Stephenson , A. A. El‐Zoka , J. R. Mianroodi , M. Rohwerder , B. Gault , D. Raabe , Acta Mater. 2021, 212, 116933.

[90] M. A. Pellow , C. J. M. Emmott , C. J. Barnhart , S. M. Benson , Energy Environ. Sci. 2015, 8, 1938.

[91] Global Hydrogen Fueling Station Market Outlook to 2027, https://www.blackridgeresearch.com/reports/global‐hydrogen‐refueling‐stations‐hrs‐market, (accessed: October 2022).

[92] P. O. for the, L. Environment, Climate and Energy Outlook 2020, 2020.

[93] CO₂ and, Greenhouse Gas Emissions – Our World in Data, https://ourworldindata.org/co2‐and‐greenhouse‐gas‐emissions, (accessed: October 2022).

[94] THyGA | Testing Hydrogen admixture for Gas Applications, https://thyga‐project.eu/, (accessed: October 2022).

[95] K. Zhang , X. Liang , L. Wang , K. Sun , Y. Wang , Z. Xie , Q. Wu , X. Bai , M. S. Hamdy , H. Chen , X. Zou , Nano Res. Energy 2022, 1, e9120032.

